# Unifying low-level mechanistic and high-level Bayesian explanations of bistable perceptions: neuronal adaptation for cortical inference

**DOI:** 10.1186/1471-2202-12-S1-P320

**Published:** 2011-07-18

**Authors:** David P Reichert, Peggy Series, Amos J Storkey

**Affiliations:** 1 School of Informatics, University of Edinburgh, Edinburgh, EH8 9AB, UK

## 

Ambiguous images such as the Necker cube evoke bistable perceptions in observers, where the conscious percept alternates between the two possible image interpretations. One classic explanation is that mechanisms like neuronal adaptation underlie the switching phenomenon [1]. On the other hand, one possible high-level explanation [2] is that in performing Bayesian inference, the brain might explore the multimodal posterior distribution over possible image interpretations. For example, sampling from a bimodal distribution could explain the perceptual switching [2], and probabilistic sampling might be a general principle underlying cortical inference [3]. In this computational study of bistable perceptions, we show that both views can be combined: Neuronal adaptation such as changes of neuronal excitability and synaptic depression can be understood to *improve* the sampling algorithm the brain might perform.

We use Deep Boltzmann Machines (DBMs) as models of cortical processing [4]. DBMs are hierarchal probabilistic neural networks that learn to generate or predict the data they are trained on. For doing inference, one can utilize Markov chain Monte Carlo methods such as Gibbs-sampling, corresponding to the model's neurons switching on stochastically. The model then performs a random walk in state space, exploring the various learned interpretations of an image, thus potentially explaining bistable perceptions (cf. [5]). However, in machine learning one often finds that exploring multi-modal posterior distributions in high-dimensional spaces can be problematic, as models can get stuck in individual modes ('the Markov chain does not mix'). Very recent machine learning work [6,7] has devised a class of methods that alleviate this issue by dynamically changing the model parameters, the connection strengths, during sampling. Interestingly, Welling [6] suggested a potential connection to dynamic synapses in biology.

Here, we make this connection explicit. Using a DBM model that has learned to represent toy images of unambiguous cubes, we show how a sampling algorithm similar to [7] can be understood as modeling dynamic changes to neuronal excitability and synaptic strength, making it possible to switch more easily between modes of the posterior distribution, i.e. the two likely interpretations of the ambiguous Necker cube. Unlike [2], who design an ad-hoc abstract inference process, our approach is based on a concrete hierarchical neural network that has learned to represent the images, and utilizes canonical inference methods, with the additional twist of relating the latter to neuronal adaptation. We also make different hypotheses than [2] w.r.t. where in the brain the perceptual switch is realized (namely, gradually throughout the visual hierarchy) and how probability distributions are represented (one sample at a time). Our study naturally follows up on our earlier work [4], where we showed how similar, homeostatic mechanisms on a slower timescale can cause hallucinations. As a final contribution, we demonstrate how spatial attention directed to specific features of the Necker cube can influence the perceptual switching [8].
